# Genome wide identification of chilling responsive microRNAs in *Prunus persica*

**DOI:** 10.1186/1471-2164-13-481

**Published:** 2012-09-15

**Authors:** Abdelali Barakat, Aditya Sriram, Joseph Park, Tetyana Zhebentyayeva, Dorrie Main, Albert Abbott

**Affiliations:** 1 Department of Biochemistry and Genetics, Clemson University, Clemson, SC, 29631, USA; 2 Department of Computer Sciences, Clemson University, Clemson, SC, 29631, USA; 3 Department of Biochemistry and Molecular Biology, Pennsylvania State University, University Park, PA, 16802, USA; 4 Department of Horticulture and Landscape Architecture, Washington State University, Pullman, WA, 99164-6414, USA

**Keywords:** microRNAs, Distribution, Expression, Cold stress, Chilling requirement, Bud development

## Abstract

**Background:**

MicroRNAs (miRNAs) are small RNAs (sRNAs) approximately 21 nucleotides in length that negatively control gene expression by cleaving or inhibiting the translation of target gene transcripts. Within this context, miRNAs and siRNAs are coming to the forefront as molecular mediators of gene regulation in plant responses to annual temperature cycling and cold stress. For this reason, we chose to identify and characterize the conserved and non-conserved miRNA component of peach (*Prunus persica* (L.) Batsch) focusing our efforts on both the recently released whole genome sequence of peach and sRNA transcriptome sequences from two tissues representing non-dormant leaves and dormant leaf buds. Conserved and non-conserved miRNAs, and their targets were identified. These sRNA resources were used to identify cold-responsive miRNAs whose gene targets co-localize with previously described QTLs for chilling requirement (CR).

**Results:**

Analysis of 21 million peach sRNA reads allowed us to identify 157 and 230 conserved and non-conserved miRNA sequences. Among the non-conserved miRNAs, we identified 205 that seem to be specific to peach. Comparative genome analysis between peach and *Arabidopsis* showed that conserved miRNA families, with the exception of miR5021, are similar in size. Sixteen of these conserved miRNA families are deeply rooted in land plant phylogeny as they are present in mosses and/or lycophytes. Within the other conserved miRNA families, five families (miR1446, miR473, miR479, miR3629, and miR3627) were reported only in tree species (*Populustrichocarpa*,* Citrus trifolia*, and *Prunus persica*). Expression analysis identified several up-regulated or down-regulated miRNAs in winter buds versus young leaves. A search of the peach proteome allowed the prediction of target genes for most of the conserved miRNAs and a large fraction of non-conserved miRNAs. A fraction of predicted targets in peach have not been previously reported in other species. Several conserved and non-conserved miRNAs and miRNA-regulated genes co-localize with Quantitative Trait Loci (QTLs) for chilling requirement (CR-QTL) and bloom date (BD-QTL).

**Conclusions:**

In this work, we identified a large set of conserved and non-conserved miRNAs and describe their evolutionary footprint in angiosperm lineages. Several of these miRNAs were induced in winter buds and co-localized with QTLs for chilling requirement and bloom date thus making their gene targets potential candidates for mediating plant responses to cold stress. Several peach homologs of genes participating in the regulation of vernalization in *Arabidopsis* were identified as differentially expressed miRNAs targets, potentially linking these gene activities to cold responses in peach dormant buds. The non-conserved miRNAs may regulate cellular, physiological or developmental processes specific to peach and/or other tree species.

## Background

microRNAs (miRNAs) are small non-coding RNAs approximately 19 to 22 nucleotides (nt) in length that function in gene regulation at the post-transcriptional level [1]. They are transcribed from one DNA strand as long precursors, which under the processing of various enzymes lead to mature double strand miRNAs [2]. One strand of the miRNA is degraded; the other is incorporated in a ribonucleoprotein complex, the RNA-induced silencing complex (RISC), which is involved in RNA interference (RNAi). The mature miRNA guides the action of RISC to degrade mRNAs of its targets.

Research in recent years demonstrated that miRNAs play a significant role in the epigenetic regulation of genes controlling various cellular and developmental processes [3]. Indeed, most miRNA targets identified to date are involved in the development of plant organs such as root, leaf, and flower [1,3]. However, a significant fraction of miRNA target genes seems to be involved in cellular defense against abiotic stresses such as nutrient deprivation, drought, heat, and UV exposure [4] or biotic stresses including attack by fungi such as *Phakopsora pachyrhizi* and *Cronartium quercuum*[5,6] , or bacteria such as *Pseudomonas syringae*[7].

Cold temperature exposure is a major abiotic stress that affects plant development and growth and significantly impacts crop production. Plants evolved various processes to cope with cold stress at the cellular and developmental levels. Flower and vegetative winter bud dormancy responses enable plants to avoid flower and leaf damage caused by fluctuating temperatures during winter and to synchronize the re-initiation of growth with the annual return of optimal temperatures in the spring. Genetics analyses of the cold response linked with studies on vernalization suggest that sRNAs play a significant role in these phenological traits. For instance, expression analysis in *Arabidopsis thalianaPopulus trichocarpaBrachypodium distachyon* and other plant species [4,8-10] identified several miRNAs with altered expression under cold stress. Another study showed that *Arabidopsis* miR402 positively regulates seed germination under dehydration or cold stress conditions [11] by repressing DEMETER-LIKE protein3 (DML3), which is involved in DNA demethylation [12]. Other studies [13,14] showed that the repression of the *Flowering LocusC (FLC)* by vernalization was mediated by a 24-nt siRNA.

Peach belongs to *Prunoideae*, one of the four subfamilies of the *Rosaceae*[15]. Due to the compact genome size (227 Mb) and the extensive genomics and genetics resources available for this species (e.g. a high density integrated genetic/physical map, extensive cDNA sequence resources, availability of a doubled haploid), the peach has been highlighted as a genome model for fruiting trees in the *Rosaceae*. In addition, as a temperate perennial tree species with a reasonably short juvenility period (two years) and a reasonably diverse breeding germplasm, it represents an excellent model for the genetic dissection of traits important to both basic and applied tree research. As a stone fruit, peach holds a key phylogenetic position in the *Rosaceae*, which is comprised of over 100 genera and 3,000 species [15]. The *Rosaceae* family ranks third in economic importance of the plant families in temperate regions [16]; it includes many species such as almond, apple, apricot, blackberry, cherry, peach, raspberry, rose and strawberry, valued for nuts, fruits and flowers. Recently the genome of the peach was completely sequenced and the assembly and gene annotation made publicly available on the Genome Database for *Rosaceae*[17,18]. However, no study reporting miRNAs from peach is yet published. Identifying miRNAs from peach is very important to infer ancestral relationships from divergent pools of miRNAs in other closely related *Rosaceae* species [19,20] and other more distantly related angiosperms. Moreover, the identification of miRNAs from peach whose expression is correlated with changing environmental cues provides a tool for uncovering the network of regulated gene activities associated with phenological responses in trees. Identifying miRNAs from this species enables analyses of the function of these miRNAs in *Rosaceae* species and the evaluation of the level of conservation of the miRNA-mediated gene regulatory networks in this botanical family.

In this work, sRNAs from young emerging leaves and chilled vegetative buds were sequenced using the SOLID Platform (Applied Biosystems, Foster City, CA) and the sRNA transcriptome was analyzed. sRNA analysis in combination with miRNA mining in the whole genome sequences of peach identified 157 and 230 unique conserved and non-conserved miRNA sequences, respectively. *In silico* expression analyses enabled the identification of several miRNAs displaying differential expression in winter dormant buds versus pre-dormant leaves. The prediction of miRNA targets showed that these miRNAs targets are involved in various biological processes. Several miRNAs or miRNA-regulated genes were found co-localizing with previously reported QTLs for chilling requirement (CR-QTLs) and bloom date (BD-QTLs) [21]. Several of these genes appear to be involved in response to cold stress. Comparative and phylogenetic analysis of miRNA distribution in peach and other species provide insight into the evolution of miRNA families.

## Results

### Analysis of sRNAs from leaves and dormant buds

**Figure 1:**
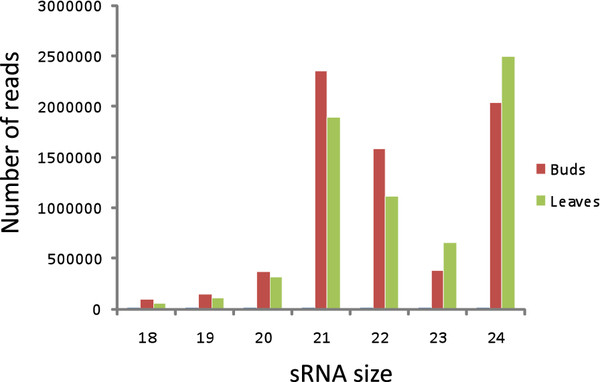
Size distribution of sequenced small RNAs ranging between 18 and 24 nt.

A total of 21 million sequences were generated from leaves and bud samples using the ABI SOLID sequencing platform. The number of sRNA reads identified at each step of the miRNA prediction pipeline is summarized in Table 1. Among these sequences, 10,151,770 and 10,899,501 were from leaves and buds, respectively, with sizes ranging from 18 and 24 nt; with two majors peaks at 21 and 24 nt (Figure 1). Among the, 47,944 and 65,413 reads from leaves and buds, respectively, were contaminant rRNAs, tRNAs, and snoRNAs. We removed 557,659 and 992,999 reads from leaves and buds, respectively, that had more than ten matches to the peach genome, as these likely correspond to sRNAs from repeated sequences. Querying the remaining sequences against miRBase (version 17) [22] identified 22,984 and 28,827 read matches from leaves and buds, respectively. The remaining 6,009,367 (1,093,920 unique reads) and 7,384,277 (236,938 unique reads) sequences from leaves and buds, respectively, corresponded to other sRNAs including non-conserved miRNAs. After removing length variants, final sets of 478,393 and 210,296 reads corresponding to non-conserved miRNAs from leaves and buds, respectively, were identified.

**Table 1:** Sequencing summary of peach sRNAs

**small RNAs**	**Leaves**	**Winter buds**
18-24 nt (reads)	10,151,770	10,899,501
Contaminant (reads)	47,944	65,413
More than 10 hits (reads)	557,659	992,999
Conserved miRNAs (reads)	22,984	28,827
Non-conserved sRNA sequences (reads)	6,009,367	7,384,277
Non-conserved sRNA unique sequences (reads)	478,393	210,296
Non-conserved sRNAs passed MirCheck and have miRNA* prior to visual inspection and length variant removal (reads)	19,993	7326

### Identification of conserved miRNAs

**Figure 2:**
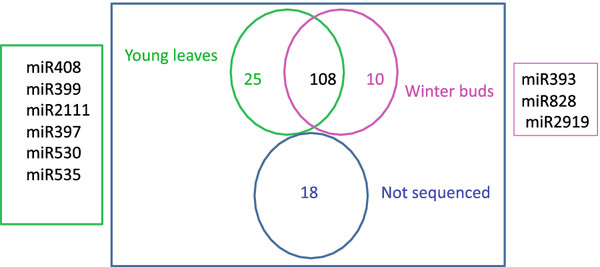
Venn diagram representing the number of conserved and non-conserved miRNAs identified from either leaves, buds, or both samples.

**Figure 3:**
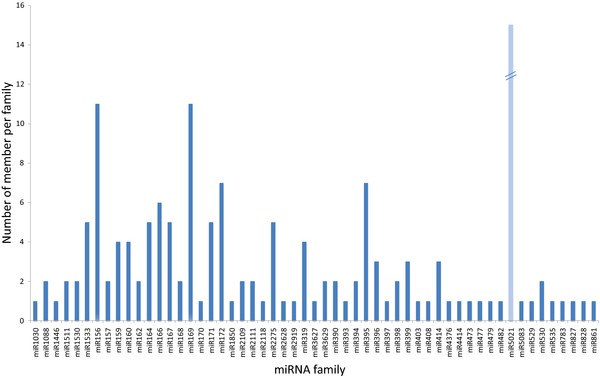
Distribution of conserved miRNA family size in peach.

**Figure 4:**
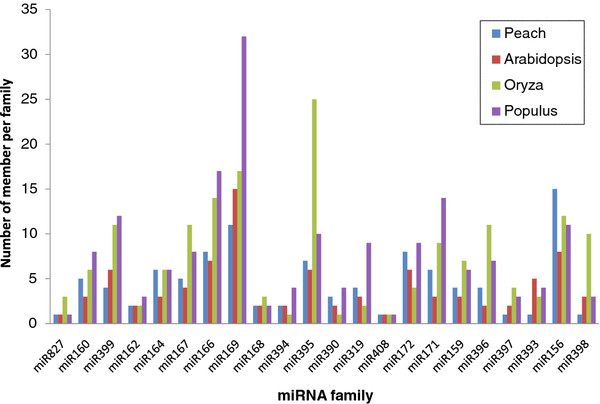
**miRNA family size distribution in model species.** Number of miRNA paralogs identified in peach in this study versus *Arabidopsis*, *Populus*, and *Oryza*, from 15 conserved miRNA families. Note the extension of the size of miR5021 in peach.

22,984 and 28,827 reads from buds and leaves have at least one match with less than two substitutions to conserved miRNAs from miRBase. Larger read numbers (80,422 and 87,858 from buds and leaves, respectively) were identified when sRNA sequences were mapped to the genome using Bowtie [23]. However, the numbers of unique sequences obtained using Bowtie or direct translation of color space to nt format using a home made script were similar. Precursors of these miRNAs were retrieved from the peach genome sequence, folded and checked for miRNA features. miRNAs were annotated using criteria described by Meyers and collaborators [24]. We first checked the biogenesis criterion, i.e. precise excision from the stem of a stem-loop precursor. In the absence of expression for individual miRNA* sequences, evidence of expression in both bud and leaf libraries or a strong accumulation in one of the libraries was used as evidence for the validity of the miRNA*. In the absence of expression of the miRNA and the miRNA*, other criteria such as conservation of both miRNA sequences and stem-loop secondary structure with other well characterized miRNAs in other species, and the existence of well annotated member of the same miRNA family in peach were used to validate the miRNA. These analyses led to the identification of 157 unique sequences that belong to 57 conserved miRNA families (Figure 2 and Additional file 1: Table S1). miRNA* sequences were identified for 101 (62.73 %) of the 157 identified miRNA sequences (Additional file 1: Table S1). Of the 60 miRNAs with no sequenced miRNA*, 36 were members of the peach miRNA families of which at least one member was confirmed to produce miRNAs by the biogenesis criteria. The remaining 25 were annotated as miRNAs based on fold back and other ancillary criteria. Ten and nine of these miRNAs were present in the sequences from leave and bud libraries, respectively, or showed a strong accumulation in one of the libraries. Six were annotated based on the conservation of their stem-loop secondary structure and the mature miRNA sequence with previously characterized miRNA sequences. Of the 157 sequences, 133 miRNAs were identified in both leaves and buds. 25 and 11 tissue specific miRNAs were identified from leaves and buds, respectively. They are indicated in Additional file 1: Table S1 by a number of reads labeled “0”. Comparison of family sizes showed that the number of unique sequences (miRNA members) varied widely between miRNA families. The number of members per miRNA family ranged between one and 18 (Additional file 1: Table S1 and Figure 3). miRNA families such as miR156, miR169, miR172, miR395, and miR5021 have the largest number of members with the latter having 18 members. When searching the peach genome for homologs of miR5021, 28 additional members were identified. Several of them were expressed in both libraries and satisfied the folding criteria, but no miRNA* sequences were evident. Search of these sequences against peach repeated sequences did not show any significant hits. The large number of members identified for this family, suggested that these miRNAs may originate from repeated sequences, thus, we decided to not include members for which no miRNA* sequences were present. Most of conserved families common to *Arabidopsis* and peach (miR156, miR159, miR160, miR164, miR166, miR171, miR172, miR319, miR390, miR395, and miR396) did not show significant size variation (Figure 4). Furthermore, the size of miR5021 was expanded in peach. Analyzing the distribution in the genome showed that the miRNAs identified in this study were distributed on all eight chromosomes of peach with more miRNA genes on chromosome 1 (g test, P value = 0.4522). Few miRNAs were located on scaffold 9 that has not yet been assigned to a chromosome.

### Distribution of peach conserved miRNAs in land plant species

All miRNAs that are conserved in *Arabidopsis*, rice and *Populus* (11) or conserved between *Arabidopsis* and *Populus* (23) were found in the peach sequences (Additional file 2: Table S2). Sixteen of these miRNA families were deeply rooted in the phylogeny of land plants; they were present in either moss or lycophytes (Additional file 2: Table S2). Among these miRNA families, five (miR1446, miR473, miR479, miR3629, and miR3627) were reported only in tree species (*Populustrichocarpa*, *Citrus trifolia*, and *Prunus persica* (L.) Batsch). A search for these miRNAs in the genomes of *Arabidopsis* and *Oryza* using patscan assembly with up to two substitutions identified a homolog only for miR3629 in *Oryza*. Twenty three conserved miRNA families were found in peach and other plant species but have not been reported in other tree species. A search of these miRNA in the *Populus* genome enabled the identification of homologs from 11 families (Additional file 1: Table S1). Other miRNA families (miR1030 and miR1088) were reported only in moss (*Physcomitrella patens*) or fern (*Selaginella moellendorfii*) and peach. A set of miRNA (miR2275, miR1850, miR2919, and miR5083) were found in peach and monocots but were not reported in other land plant species.

Mapping the identified conserved miRNAs on previously reported CR-QTLs and BD-QTLs [21] showed that 37 sequences belonging to 18 conserved miRNA families were co-localizing with CR-QTLs and BD-QTLs. Three of these families (miR5021, miR2919, and miR414) were predicted to target genes involved in response to cold stress.

### Identification of non-conserved miRNAs

MirCheck [1] analysis of 478,393 and 210,296 non-conserved sRNA sequences from leaves and buds, respectively, enabled the identification of 19,993 and 7326 unique miRNA sequences that satisfied the biogenesis criterion. After removing miRNA candidates that have no sequenced miRNA* and those that were represented by less than two reads in either leaves or buds datasets, as well as length variants, 230 unique non-conserved miRNAs were identified (Additional file 3: Table S3). A search of these sequences that have not been previously reported in other model plants using patscan with up to two substitutions showed that 15, 14, and 22 were found in *ArabidopsisOryza*, and *Populus*, respectively. These non-conserved miRNAs were represented by a number of reads ranging from 1 to 5636 and from 1 to 3446 for buds and leaves respectively. Among the identified 230 non-conserved miRNAs, 31 were co-localized with CR-QTLs and BD-QTLs and five of them regulate genes involved in development processes. Six of these miRNAs (pper-3-1, pper-49-1, pper-71-1, pper-99-1, pper-161-1, and pper-164-2) target genes involved in development while two others (pper-99-1 and pper-49-1) regulate genes involved in cold response. One miRNA (pper-82-1) of these 31 was also differentially highly expressed in winter buds (see below).

### Expression profiling of miRNAs in leaves and dormant buds

**Figure 5:**
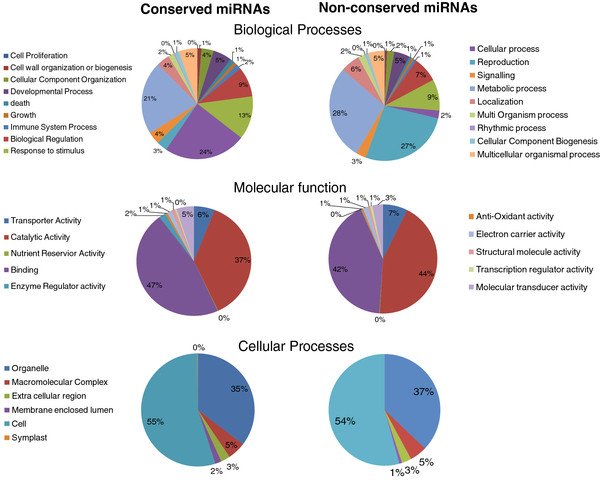
**Pie chart representation of Gene Ontology classification of putative molecular functions of the predicted targets ofpeachmiRNAs as well as biological and cellular processes in which they are involved.**

Expression of conserved and non-conserved miRNAs was assessed by counting the number of their corresponding reads in leaves and buds datasets. The expression varies widely between miRNAs from different families or between members from the same family within the same tissues or between leaves and buds (Additional file 4: Table S4 and Additional file 5: Table S5). The numbers of reads vary between one to 7146 and one to 7104 for buds and leaves, respectively. miR156, miR159, miR166, miR172, miR390, miR396, and miR5021 are the most expressed families in bud tissues. Similar sets of miRNAs, with the exception of miR167 and miR395, were highly expressed in leaves. In silico expression analyses of miRNAs using DEGseq [25] identified 19 sequences belonging to eight conserved miRNA families (miR156, miR157, miR164, miR172, miR393, miR396, miR414, and miR2275) induced in winter buds versus leaves (Additional file 6: Table S6). When DEGseq analyses were run using the number of reads corresponding to the most frequent length variant, similar miRNA families were found to be highly differentially expressed in buds. On the contrary, 41 sequences belonging to 18 conserved miRNA families were highly expressed in leaves (Additional file 4: Table S4). Comparative *in silico* expression analysis of non-conserved miRNAs using DEGseq showed that 10 non-conserved miRNAs (pper_82_1, pper_93_1, pper_98_1, pper_111_1, pper_111_2, pper_111_3, pper_143_4, pper_143_2, pper_143_1, and pper_143_3) were highly differentially expressed in winter buds (Figure 5 and Additional file 5: Table S5). Contrastingly, 60 non-conserved miRNA sequences were highly expressed in leaves.

### Prediction of conserved and non-conserved miRNA targets

A target search using the peach proteome [18] enabled the prediction of targets for 136 out of 157 (86.6 %) conserved miRNAs (Additional file 7: Table S7 and Additional file 8: Table S8). At least one target was identified for each miRNA family; however, several targets were identified for most miRNAs. Targets with top scores are presented in Additional file 7: Table S7. The other targets identified with scores less than four are listed in Additional file 8: Table S8. GO annotation using the *Arabidopsis* proteome (Figure 5) showed that 12.64 % and 23.94 %, and 21.39 % of the targets belong to the function categories “response to stimuli”, “cellular process” and “development processes” respectively. Genes from development processes are all involved in various processes of flower bud induction and flower morphogenesis and development. A few genes such as *chitinase A*, and *AICARFT/IMPCHase* are involved in regulation of plant response to cold stress. A large fraction of the predicted miRNA targets (Additional file 8: Table S8) were previously identified in other studies. However, new targets were predicted for several conserved miRNA families in peach. Confirmation of these predicted targets by analyzing the degradome [26] or using 5’ RACE is necessary.

At least one target gene was predicted for 166 out of 230 (72.1 %) non-conserved miRNAs (Additional file 9: Table S9 and Additional file 10: Table S10). GO annotation analysis identified several genes involved in plant development including several *embryo defective proteins*, *Late embryogenesis abundant*, *PPR2*, and *DNA glycosylase*. A few targets encode proteins involved in cold stress response such as Annexins, WD-40, phosphatidylinositol-specific phospholipase C, and cellulose synthase. Most importantly, several identified targets encode proteins involved in sRNA biogenesis and methylation such as a Dicer homolog (RNA helicase), ribonuclease III, DNA glycosylase DEMETER, histone 3 lysine 9 specific methyltransferase, and SUVH4/KYP homolog.

Several genes from QTL regions were found being targeted by conserved and non-conserved miRNAs. One of these genes (*Reduced Vernalization 1* (*VRN1*) is a key player in regulating vernalization in other plant species. It was predicted to be targeted by a non-conserved miRNA (pper_100_1).

## Discussion

### Conserved miRNAs and distribution in angiosperm lineages

sRNA sequencing of over 21 million sRNA reads from predormant young leaves and dormant leaf buds enabled the identification of over 387 conserved and non-conserved miRNA sequences from peach. The conserved miRNA dataset include all miRNAs conserved in *ArabidopsisOryza* and *Populus* or conserved between *Arabidopsis* and *Populus* suggesting that the dataset identified here is complete. Among the identified conserved miRNA set, five conserved miRNA families (miR1446, miR473, miR479, miR3629, and miR3627) and several non-conserved miRNAs were reported only in tree species (*PopulustrichocarpaCitrus trifolia*, and *Prunus persica* (L.) Batsch). A search of these miRNAs in the genomes of the herbaceous species *Arabidopsis* and *Oryza* confirmed that four of them were not found in the *Arabidopsis* and *Oryza* genomes. These results confirm that a small set of miRNAs may exist only in tree species and function in biological processes specific to them. For instance, it was suggested that miR1446 and miR473 may play important roles in the growth of trees such as the formation of specialized woody tissue or in the response of trees to mechanical stress [9]. Other miRNA families (miR1030, miR1088) were reported only in moss (*Physcomitrella patens*) or fern (*Selaginella moellendorfii*) and peach. A set of miRNAs (miR1850, miR2275, miR2919, miR2931 and miR5083) was found in peach and monocots but not in other land plant species. This pattern of gain/loss of certain miRNA families in various plant lineages was previously reported [27,28]. The inability to detect some miRNAs in some angiosperm lineages could be due to the fact that these miRNAs were not expressed in tissues used for constructing sRNA libraries in the non-model species studied. However, the absence of these miRNAs in whole genome sequences of model species could only be explained by the fact that some miRNAs, such as the ones identified only in trees, are specific to some lineages or species. It is noteworthy that the loss of some miRNAs is not unidirectional as some miRNAs from peach were lost either in monocots (*Oryza*), or eudicots (*Arabidopsis* and *Populus*).

Analysis of the size of miRNA gene families showed that most of the conserved miRNA families from peach have a similar number of members in *Arabidopsis* but less genes than in *Populus*. However, one miRNA family (miR5021) was over-represented in peach compared to both *Arabidopsis* and *Populus*. The increase in gene number of this family could be associated with the biological role of these miRNAs in peach.

### miRNAs co-localizing with CR-QTLs and BD-QTLs

Since we were interested in CR, we searched for miRNAs that co-localize with CR-QTLs and BD-QTLs. We found 31 miRNAs co-localizing with seven CR-QTLs and BD-QTLs. Members of four families (miR5021, miR164, miR414, miR396, miR2919) target genes encoding proteins involved in dormancy such as embryo-defective or maternal effect embryo arrest 14 (MEE14). Others encode proteins involved in cold stress response such as annexin 5, AICARFT/IMPCHase, ATMEKK1, cellulose, and MAPK/ERK kinase. The list of targets also includes genes encoding a histone-lysine N-methyltransferase that is involved in vegetative to reproductive phase transition of meristem. Like conserved miRNAs, six non-conserved miRNAs were located in the CR-QTL and BD-QTL regions. Five of these miRNAs target genes that are known to be involved in development processes and two target genes known to respond to cold stress.

### miRNA expression signatures associated with chilling requirement

Relative expression of the conserved miRNAs varied widely, based on the number of sequences observed for each miRNA in our dataset. The ten most highly expressed miRNAs (miR156, miR157, miR159, miR164, miR167, miR172, miR393, miR396, miR414, miR2275, and miR5021) in buds and leaves are miRNAs regulating genes involved in flower and leaf development processes such as integument development, leaf morphogenesis, meristem initiation, maintenance, and growth, bilateral symmetry determination, organ morphogenesis, plant phase transition, shoot apical meristem identity, flower and fruit development, and plant architecture. All of these miRNAs are cold responsive [29]. Unlike conserved miRNAs, only one of the highest expressed non-conserved miRNAs in pre-dormant leaves is known to play a role in a developmental process. Expression analysis using DEGseq identified several miRNAs highly expressed in winter buds. Genes targeted by these miRNAs include genes involved in apical meristem formation, pollen development, and an RNA slider that may play a role in posttranscriptional silencing. Several of these miRNAs were previously reported as induced following cold stress [10]. The occurrence of stress-related *cis* elements such W-box in the regions of several of these miRNAs may play a role in their activation [10]. Three of these miRNA genes (miR156, miR172, and miR398) were also reported as responding to cold stress in several studies [4,8,10,29,30]. miR172 was suggested to fine-tune plant development under continuously fluctuating temperature conditions [29]. Similarly, ten non-conserved miRNAs were differentially expressed in dormant winter buds (at least a two fold higher expression) of which three were located in CR-QTLs and BD-QTLs. However, none of these miRNAs seem to regulate known genes involved in cold stress, flower or leaf development.

### Prediction of conserved and non-conserved miRNA targets

Target analysis enabled the prediction of target genes for all conserved and over half of the non-conserved miRNAs. miRNAs for which no target could be identified may correspond to young non-functional miRNAs. A large set of genes targeted by peach miRNAs encode genes involved in flower and leaf development. A small set of genes are involved in cold stress. Among these genes is *VRN1* a known regulator in vernalization [31]. This is in agreement with previous studies on *ArabidopsisBrachypodium*, and *Populus* showing that miRNAs are involved in response to cold stress [4,8-10,32]. We also identified genes involved in methylation or RNA biogenesis that may function in epigenetic regulation of plant response to cold stress. Among these genes, a *Dicer homolog (RNA helicase)* involved in miRNA processing [33], a *ribonuclease III* that is required for endogenous RDR2-dependent siRNA formation [34], a *DNA glycosylase DEMETER* responsible for endosperm maternal-allele-specific hypomethylation at the *MEDEA (MEA)* gene [35], a *histone 3 lysine 9 specific methyltransferase* involved in the maintenance of DNA methylation [36], and a *SUVH4/KYP* homolog that is involved in epigenetic control of *FLC* expression [37]. This is in accordance with recent studies showing that epigenetic modifications including methylation, siRNA signatures, and histone modifications play a major role in vernalization [13,38].

Furthermore, several genes from CR-QTLs and BD-QTLs regions that were reported as playing key roles in regulating vernalization were predicted targets by miRNAs. Several of these genes belong to the polycomb repressor 2 silencing complex that regulates the expression of the key flowering gene *FLC*[38]. For instance, *VRN1* is targeted by the non-conserved miRNA (pper_100_1). These two genes function in maintaining the repression of the major target of the vernalization pathway, the floral repressor *FLC*. However, it is currently unknown what role these two genes may play in CR regulation in perennial trees, which is a significantly different phenological character particularly since the *FLC* gene has not yet been reported as present in the peach genome or the genomes of its close relatives (Zhebentyayeva, personnal communication).

## Conclusions

In conclusion, this study identified hundreds of conserved and non-conserved miRNAs and their target genes in peach, a *Rosaceae* model species expanding our understanding of the pattern of conservation of miRNA in land plant lineages. This study also identified several cold-responsive miRNAs and their predicted gene targets present in CR-QTLs and BD-QTLs thus, potentially connecting miRNA regulatory activities to CR and dormancy in peach. This miRNA dataset will be very useful to the scientific community working on *Rosaceae* and other plant families for functional analyses of genes of interest and for deciphering the gene regulatory networks of CR and bud dormancy in peach.

## Methods

### Tissue collection and RNA preparation

Young emerging leaves and chilled vegetative winter buds (600 chill-hours accumulated) of the double haploid reference genome genotype “Lovell” (Clemson University) were used for sRNA sequencing. Samples were collected from trees grown at Musser Farm, Clemson SC, frozen in liquid nitrogen and stored at −80˚. Total RNA was prepared by the method of Carra and collaborators [39]. The quality of total RNA was assessed with an Agilent Technologies 2100 Bioanalyzer (Agilent Technologies) for possible degradation. Total RNAs from both samples used in this study have RNA Integrity Numbers (RNI) values of 7.9 and 8 indicating their high quality [40].

### sRNA libraries construction and sequencing

sRNA enrichment was performed using FlashPage according to the manufacturer’s instructions (Ambion/Life Technologies). sRNA libraries were prepared from enriched sRNA using the “Prepare Small RNA Libraries “protocol from the SOLID Total RNA-Seq Kit according to the manufacturer’s instructions (Applied Biosystems/ LifeTechnologies). cDNA samples were subjected to ePCR to prepare templated beads. Templated beads were deposited on a quarter of a flow cell and 50 nt sequencing was performed on a SOLID 3 Plus System (Applied Biosystems/Life Technologies) at Penn State University.

### sRNA analysis and identification of peach miRNAs and their targets

The sequences produced by SOLID sequencing, which correspond to sRNA-derived cDNAs, were processed using two approaches. The first one consisted of converting sequences from color space to nucleotide format using custom perl scripts. The second one consisted in mapping sequences in color space format to the peach genome using Bowtie software [23] and by tolerating up to two mismatches. Sequences obtained using both approaches were analyzed separately. sRNA sequences smaller than 18 and larger than 23 nucleotides in length were discarded. sRNA sequences that passed the adapter check and size filter were then screened using BLASTN against chloroplast and mitochondrial genomes [41], tRNA [42], rRNA [43], snoRNA [44], and all contaminating rRNA, tRNA and snoRNA sequences were removed. The cleaned sequences were then sorted by sequence identity, the size variants identified, and the relative count of each miRNA was determined. Unique sRNA sequences were queried against known miRNAs using the program Patscan [45] with default parameters and two mismatches in order to identify homologs of known miRNAs. Flanking sequences (150 nt at each side) of the mature miRNA were retrieved and folded using the RNA fold program [46]. The folded sequences were then input into the MirCheck program [1] to check their secondary structures. Sequences that passed MirCheck were then inspected manually for miRNA features. Annotation of miRNAs was performed as described by Meyers and collaborators [24]. Another set of miRNAs not expressed in leaf and bud datasets was identified by querying miRBase plant sequences against the peach genome using Patscan as described above. Sequences that passed MirCheck were checked for the conservation of their mature miRNA sequences and their stem loop secondary structure in other species as described previously [24]. Sequences that satisfied this criterion were also annotated as miRNAs.

Sequences with no similarity to known miRNA sequences were used to search for non-conserved miRNAs in the peach genome using Patscan with a setting of 0 mismatches, 0 insertions, and 0 deletions. Sequences that had over than 10 matches to the peach genome may correspond to repeated sequences and were removed. Genomic regions (150 at each side) harboring sRNA sequences were then retrieved and the sequences folded using RNAfold [46] and the secondary structure was checked for miRNA features using MirCheck. Sequences that passed MirCheck were sorted for redundancy. When several length variants of the same miRNA were sequenced, only variants with the highest representation were considered. Only sRNAs that passed the MirCheck filter and have a miRNA* sequenced at least in one of the libraries was annotated as miRNAs.

A search for miRNA target genes was performed by querying the peach predicted coding DNA sequences (CDS) [18] using the scoring approach previously described [47]. Only hits with a score less than 4 were considered good target sequences. Target sequences were then annotated using the *Arabidopsis* proteome (BLASTX, e-value < 1e^-5^[48].

### miRNA *in silico* expression analysis

Expression of miRNAs in leaves and buds samples were quantified using DEGseq [25]. DEGseq uses the MA plot based method with random sampling. This method works under the assumption that RNA sequencing is random sampling and the number of reads for each gene follows a binomial distribution. Fisher exact test and likelihood ratio tests were then performed on this model to identify differentially expressed genes.

## Abbreviations

ABI, Applied Biosystems; cDNA, DNA complementary to RNA; miRNA, microRNA; nt, Nucleotide; PCR, Polymerase Chain Reaction; RCA, Rolling Circle Amplification; siRNA, Small interfering RNA; sRNA, Small RNAs; snoRNA, Small nucleolar RNA; Ta-siRNA, Trans-acting-siRNA; tRNA, Transfer RNA; rRNA, Ribosomal RNA.

## Competing interests

The authors declare that they have no competing interests.

## Authors’ contributions

AB conceived and performed the sRNA analysis study, analyzed results, wrote the manuscript, and supervised the work of AS and JP. TZ collected leaves and buds, extracted total RNA, and contributed to better delineate CR-QTLs and BD-QTLs previously published. JP contributed to curating secondary structures, and helped prepare the figures and tables. AS contributed to bio-informatics analyses and discussion of the results. The miRNA study of dormant and non-dormant tissues is an integral part of a research program conceived and funded through efforts of AA and his laboratory. DM contributed to the discussion of results and edited the manuscript. DM also contributed to the presentation of miRNAs on the Genome Database for Rosaceae. All authors read and approved the final manuscript.

## Supplementary Material

Additional file 1 **Table S1. Conserved miRNAs identified in peach.** The length (len) of each miRNA, the chromosome location (Chr), the start (start) and the end (stop) position on the chromosome of each miRNA and miRNA*sequences, the miRNA orientation (Dir), whether or not a miRNA* was observed are indicated, miRNA and miRNA* sequences, and sequence coordinates on the genome. Click here for file

Additional file 2 **Table S2. Conserved microRNA (miRNA) distribution in plant lineages.** miRNA data are a compilation from miRBase 17 and this study. Divergence dates for land plant clades is from Leebens-Mack et al. (2005). Click here for file

Additional file 3 **Table S3. Non-conserved miRNAs identified in peach.** The length (len) of each miRNA, the chromosome location (Chr), the start (start) and the end (stop) position on the chromosome of each miRNA and miRNA*sequences, the miRNA orientation (Dir), whether or not a miRNA was observed are indicated, and miRNA and miRNA* sequences. Click here for file

Additional file 4 **Table S4. Expression of conserved miRNAs in leaf and bud samples.** Number of reads in leaf (L18 to L23) and bud (B18 to B23) dataset corresponding the different length variants (18 to 23 nt) of conserved miRNAs identified in peach. The number of reads of miRNA variants and the total count are indicated. Click here for file

Additional file 5 **Table S5. Expression of non-conserved miRNAs in leaf and bud samples.** Number of reads in leaf (L18 to L23) and bud (B18 to B23) dataset corresponding the different length variants (18 to 23 nt) of non-conserved miRNAs identified in peach. The number of reads of miRNA variants and the total count are indicated. Click here for file

Additional file 6 Table S6. List of conserved and non-conserved miRNAs up-regulated or down-regulated in winter buds and leaves. Click here for file

Additional file 7 **Table S7. List of targets of peach conserved miRNAs with the best scores.** The name, the target score, peach CDS name, the *Arabidopsis* homolog to peach CDS and its annotation are indicated. Click here for file

Additional file 8 **Table S8. List of all peach genes targeted by conserved miRNAs with a score less than 4.** The name, the target score, peach CDS name, the *Arabidopsis* homolog to peach CDS and its annotation are indicated. Click here for file

Additional file 9 **Table S9. List of targets of peach non-conserved miRNAs with the best scores.** The name, the target score, peach CDS name, the *Arabidopsis* homolog to peach CDS and its annotation are indicated. Click here for file

Additional file 10 **Table S10. List of all peach genes targeted by non-conserved miRNAs with a score less than 4.** The name, the target score, peach CDS name, the *Arabidopsis* homolog to peach CDS and its annotation are indicated. Click here for file
